# A model of mucopolysaccharidosis type IIIB in pigs

**DOI:** 10.1242/bio.035386

**Published:** 2018-09-26

**Authors:** Qiang Yang, Xueyan Zhao, Yuyun Xing, Chao Jiang, Kai Jiang, Pan Xu, Weiwei Liu, Jun Ren, Lusheng Huang

**Affiliations:** 1 State Key Laboratory of Pig Genetic Improvement and Production Technology, College of Animal Science and Technology, Jiangxi Agricultural University, Nanchang 330045, China; 2 Shandong Provincial Key Laboratory of Animal Disease Control and Breeding, Institute of Animal Science and Veterinary Medicine, Shandong Academy of Agricultural Sciences, Jinan 250100, China

**Keywords:** MPS IIIB, Lysosomal storage disease, *NAGLU*, Pig model

## Abstract

Mucopolysaccharidosis type IIIB (MPS IIIB) is a rare genetic disorder caused by loss-of-function mutations in the *NAGLU* gene. Pigs are an ideal large animal model for human diseases; however, a porcine model of MPS IIIB has not been reported. We have previously generated a heterozygous *NAGLU*-deficient (*NAGLU*^+/-^) Large White boar via a transgenic approach. Here we characterized phenotypes of the F_1_ offspring of this founder to establish a pig model for MPS IIIB. qRT-PCR revealed that the *NAGLU* expression level was significantly decreased in a variety of tissues in *NAGLU*^+/−^ pigs. ELISA assays showed obvious deficiency of NAGLU and higher (*P*<0.05) glycosaminoglycan levels in multiple tissues from *NAGLU*^+/−^ pigs. *NAGLU*^+/−^ pigs grew at a significantly (*P*<0.05) slower rate than control animals (*NAGLU*^+/+^). Death, mostly sudden death, occurred at all ages in *NAGLU*^+/−^ pigs, most of which died within two years. Necropsy findings included pleural adhesions, lung shrinkage and abnormalities in the pericardium and mild hepatomegaly in *NAGLU*^+/−^ pigs. Notable pathological changes were observed in the sections of brain, liver, spleen and kidney from *NAGLU*^+/−^ pigs. Brain atrophy, ventriculomegaly, cerebellar atrophy and abnormalities in the intracerebral capsule, parietal lobes and the thalamus were also evident in *NAGLU*^+/−^ pigs. Together, *NAGLU*^+/−^ pigs show typical symptoms of human MPS IIIB patients and thus represent a novel large animal model for the disease.

This article has an associated First Person interview with the first author of the paper.

## Abstract (precis)

**Summary:** We provide a pig model for both genetic and pathological studies of human MPS IIIB, which would contribute to timely diagnoses and better treatments of this rare disease.

## INTRODUCTION

Rare diseases are those disorders that affect 0.065-0.1% of the general population, as defined by the World Health Organization (WHO). There are currently more than 5000 recognized rare diseases, most of which are heritable (Haffner et al., 2002). Most pharmaceutical firms are reluctant to risk a hefty amount of investment, both capital and time, in developing treatments for these rare diseases due to very limited market sizes. Thus the majority of these diseases are currently untreatable, leaving patients and their families to suffer (Luzzatto et al., 2015), a dire situation that urges researchers to conduct both genetic and pathological studies for timely diagnoses and better treatments of rare diseases.

Mucopolysaccharidoses (MPS) are a group of metabolic disorders caused by deficiencies in any of the 11 enzymes in the lysosome that break down glycosaminoglycans (GAGs). MPS type III (MPS III), also called Sanfilippo syndrome, is characterized by lysosomal storage of heparin sulfate (Sanfilippo et al., 1963). There are four subtypes (A, B, C and D) of MPS III. These subtypes are clinically indistinguishable but are caused by defects in four distinct genes (Valstar et al., 2008). Clinical features of MPS III are mainly neurological deteriorations (Selmer et al., 2012; Verhoeven et al., 2010; Ellinwood et al., 2003), but patients may also exhibit various clinical phenotypes such as behavioral abnormality (Valstar et al., 2010), coarse face (Champion et al., 2010), enlarged liver (Ellinwood et al., 2003; Valstar et al., 2010) and splenomegaly (Gografe et al., 2009). MPS IIIB, a rare Mendelian disease, is caused by deficiencies in N-acetyl-alpha-glucosaminidase (NAGLU), which is encoded by the *NAGLU* gene, and has geographically varied prevalence of 0.42 to 0.72 per 100,000 newborns in humans (Muenzer, 2011). More than 100 loss-of-function mutations have been identified in *NAGLU*, resulting in MPS IIIB with varied severity in humans (Valstar et al., 2008). The disease develops in a progressive nature: most affected infants are apparently normal, but later show retarded development and progressive intellectual decline, leading to severe dementia and motor disease (Valstar et al., 2008). Patients with severe disease progression die within early adulthood (Valstar et al., 2008), while patients with attenuated progression can live well into adulthood (Valstar et al., 2010).

To date, a mouse model of MPS IIIB has been generated by targeted disruption of exon 6 of the *NAGLU* gene (Li et al., 1999), and this model has facilitated studies on disease progression (Gografe et al., 2003, 2009) and treatment, especially gene therapy (Di Natale et al., 2005; Heldermon et al., 2013), of this disease. A naturally occurring canine model for MPS IIIB was reported in Schipperke dogs that had greatly reduced *NAGLU* activity along with marked elevation of glycosaminoglycans in various tissues, and had apparent neurological abnormalities (Ellinwood et al., 2003). A bovine model of MPS IIIB was found in a ‘closed herd’ in Australia. Affected bulls were homozygous for a missense mutation in *NAGLU* (Karageorgos et al., 2007). However, cattle are too large to be practically used as disease models.

Pigs are an ideal large animal model for human diseases as their physiological and pathological features are more similar to those of humans as compared to rodents, and ethic burdens are less for the use of pigs than primates and dogs. However, a pig model of MPS IIIB has not been reported. In our previous study, we obtained one transgenic founder boar via somatic cell nuclear transfer technology (Zhao et al., 2016). This individual had an insertion of one copy of an exogenous gene (*BMPR-IB*) into exon 6 of the *NAGLU* gene on pig chromosome (SSC) 12 (Zhao et al., 2016). This insertion disrupted the open reading frame of *NAGLU* and possibly resulted in a pig model of MPS IIIB. In this study, we characterized the phenotypes of the F_1_ offspring of this founder animal and confirm that those *NAGLU^+/−^* F_1_ pigs carrying one copy of loss-of-function *NAGLU* gene represent a porcine model of MPS IIIB.

## RESULTS

### PCR characterization of *NAGLU^+/−^* pigs

**Fig. 1.**
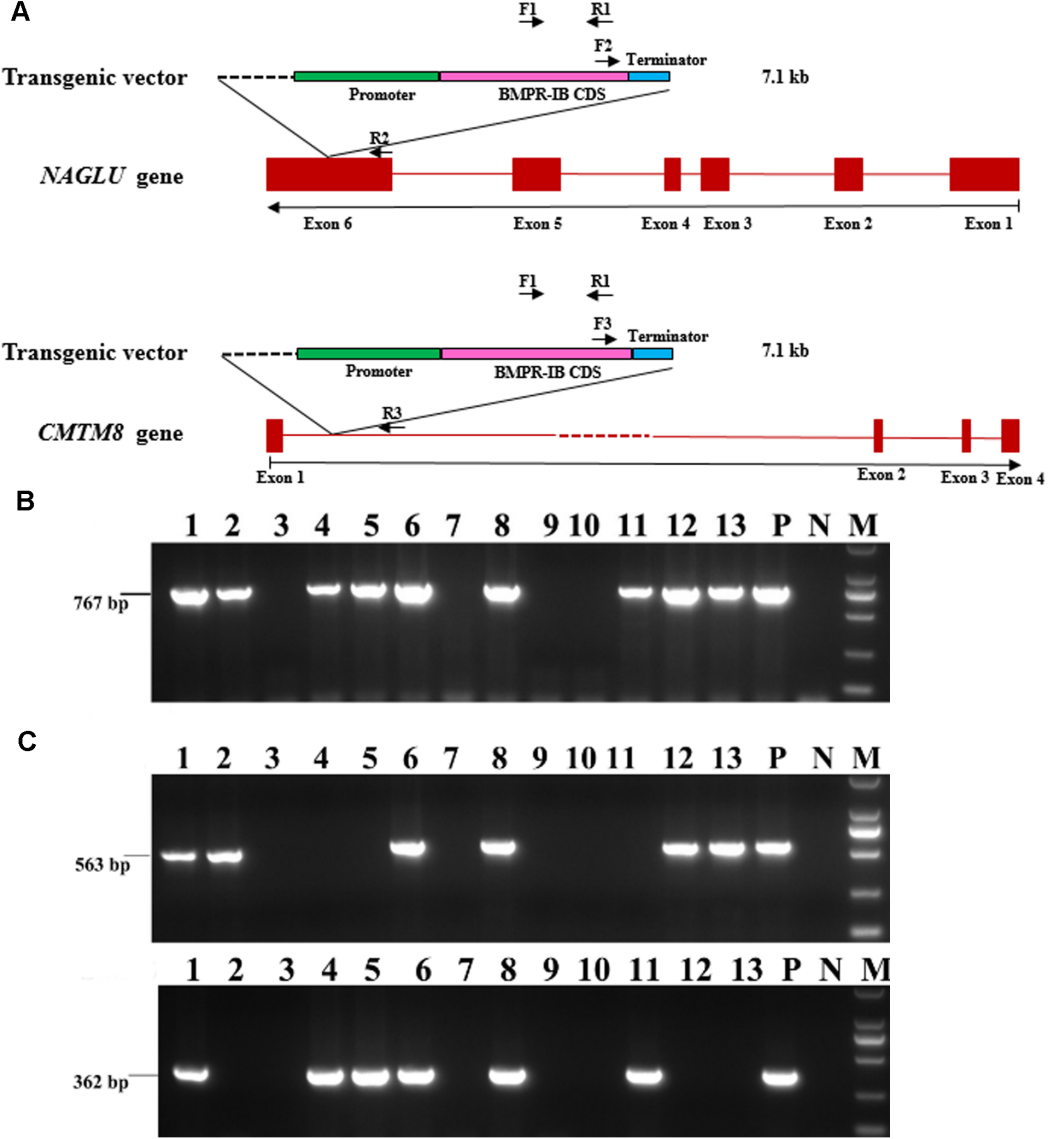
**PCR characterization of the insertion sites of the exogenous gene (*BMPR-IB*) in 92 F_1_ pigs.** (A) A diagram indicating locations of primers for genotyping of F_1_ pigs. (B) Identification of transgenic and non-transgenic pigs using primer pair F1/R1. (C) Detection of transgenic pigs that harbor insertion sites at the *NAGLU* locus on chromosome 12 (the upper one) and (or) at the *CMTM8* locus on chromosome 13 (the lower one) using primer pairs F2/R2 and F3/R3, respectively. Numbers in panels represent individuals of one litter pigs. P, the founder boar; N, water; M, 2000 bp DNA Ladder.

Of the 92 F_1_ pigs that were generated by the founder boar and seven wild-type sows, 68 were transgenic animals while 24 were non-transgenic individuals with an absence of the 767 bp amplicon via PCR using primers F1/R1 (Fig. 1A,B). The founder animal is known to have two insertion sites of the exogenous gene (*BMPR-1B*): one at the *NAGLU* locus on SSC12 and the other at the *CMTM8* locus (within the first intron of the *CMTM8* gene) on SSC13 (Zhao et al., 2016). Primers F2/R2 and F3/R3 spanning the vector and host genome (Fig. 1A,C) were used to detect the two insertion sites in these 68 transgenic F_1_ animals. We found that 22 individuals only carry the insertion site the *NAGLU* locus on SSC12, 24 pigs only harbor the insertion site the *CMTM8* locus on SSC13, and the other 22 have both insertion sites. This observation conforms to the expectation that the inserted exogenous gene is segregated in a Mendelian model in this F_1_ population.

### Effect of the inserted exogenous gene on the expression level of *NAGLU* and *CMTM8*

**Fig. 2.**
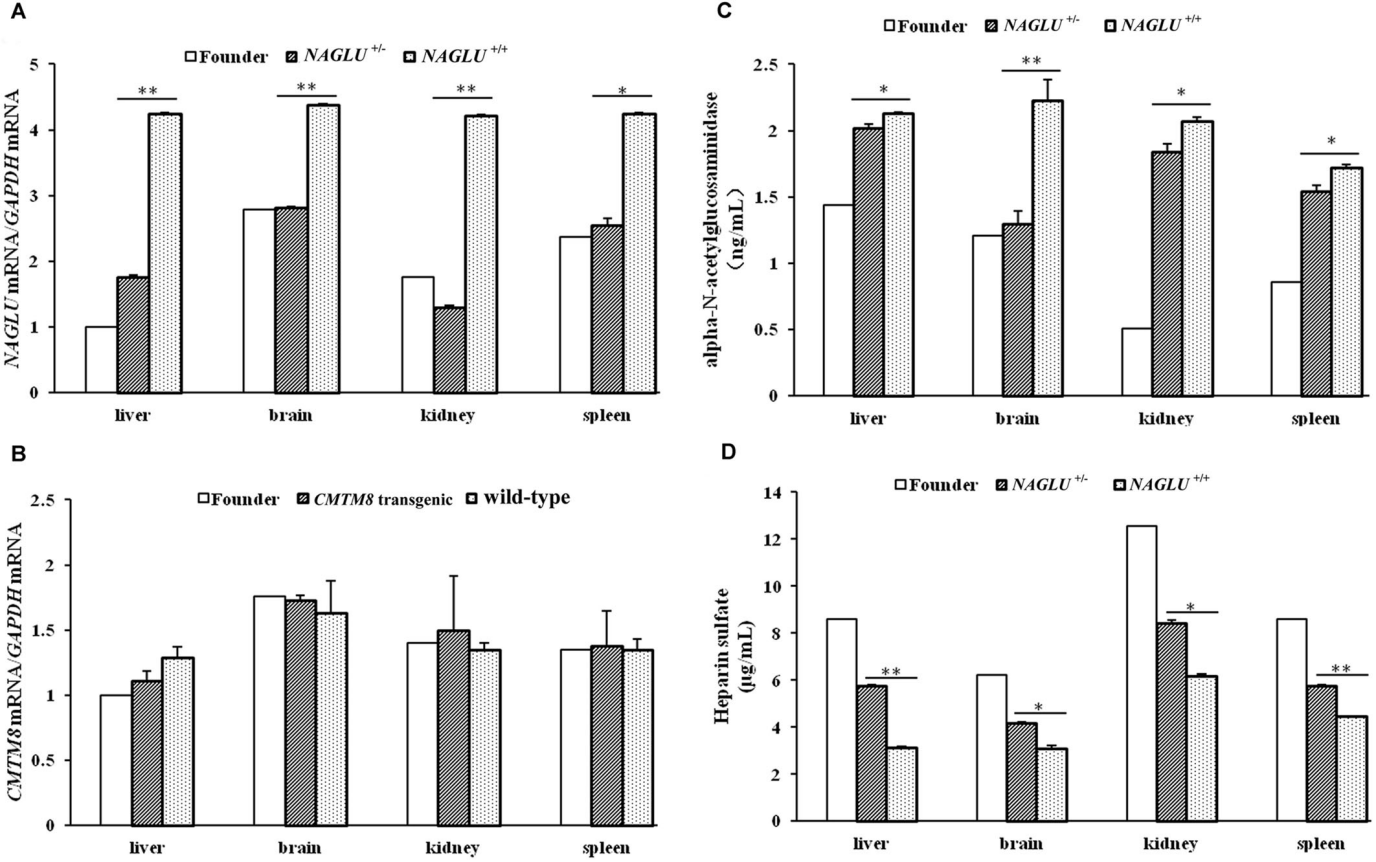
**The expression level of NAGLU and CMTM8 genes and the ELISA assays for NAGLU enzyme and HS.** (A) The mRNA level of NAGLU. (B) The mRNA level of CMTM8. (C) NAGLU enzyme amount. (D) HS storage amount. Experiments were conducted in four tissues from the 2.8-year-old founder and nine 180-day-old F1 pigs. *NAGLU*^+/−^, F_1_ pigs that carry one insertion site at the *NAGLU* locus (*n*=3); *NAGLU*^+/+^, wild-type pigs (*n*=3); *CMTM8* transgenic, F_1_ pigs that harbor one insertion site at the *CMTM8* locus (*n*=3); wild-type pigs (*n*=3). Data are presented as mean±s.e. except the founder boar. *P<0.05; **P<0.01. Phenotypic difference was not evaluated between the founder and the other individuals as only one founder animal was tested.

As the insertion site on SSC12 disrupts the sixth exon of the *NAGLU* gene and presumably affects the expression of this gene, we detected the *NAGLU* mRNA level in four tissues from three 180-day-old F_1_ pigs only harboring this insertion site (i.e. *NAGLU^+/−^* pigs) and three 180-day-old wild-type (WT) F_1_ pigs via qRT-PCR. The expression levels of *NAGLU* were significantly decreased in liver, brain, kidney (*P*<0.01) and spleen (*P*<0.05) from the *NAGLU^+/−^* pigs compared with those from the WT (*NAGLU^+/+^*) pigs (Fig. 2A). In addition, we tested the *CMTM8* mRNA level in the four tissues from three 180-day-old transgenic pigs only harboring the insertion site at the *CMTM8* locus and the three WT F_1_ pigs. No significant difference (*P*>0.05) was found in these tissues from the transgenic pigs compared with the WT individuals. The founder boar also had similar expression level of *CMTM8* mRNA in the tested tissues (Fig. 2B). The findings clearly indicate that the transgene insertion only affect the expression of the endogenous *NAGLU* but not *CMTM8* gene.

### Enzyme amount of NAGLU and accumulation of heparin sulfate in *NAGLU^+/−^* pigs

**Fig. 3.**
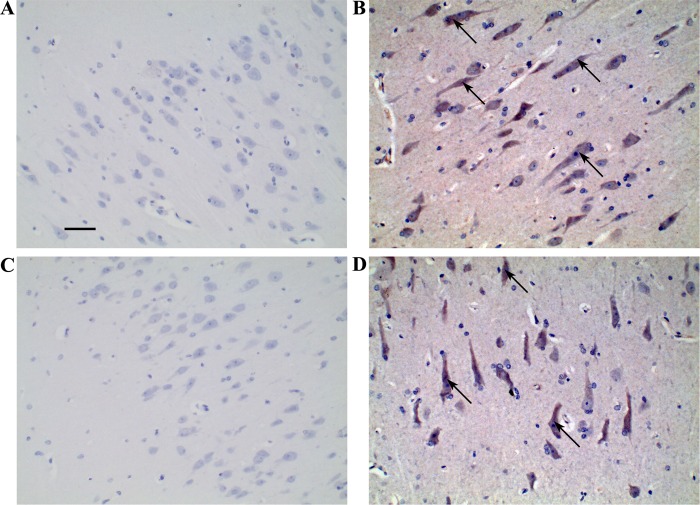
**Detection of NAGLU in the brain tissue from two *NAGLU*^+/−^ pigs and two wild-type half sibs at the age of 2.5 years using immunohistochemical staining.** (A,C) *NAGLU*^+/−^ pigs. (B,D) Wild-type pigs. Scale bar: 50 µm. Representative immunohistochemical-stained signals of NAGLU are indicated by arrows.

We detected the enzyme amount of NAGLU and storage of heparin sulfate (HS) in liver, brain, kidney and spleen tissues in the above-mentioned three 180-day-old *NAGLU^+/−^* pigs and three WT pigs via ELISA assay. We found that the enzyme amount was significantly decreased (Fig. 2C, *P*<0.05) and the accumulation of HS was remarkably increased (Fig. 2D, *P*<0.05) in all these four tissues from the *NAGLU^+/−^* pigs as compared to those from the WT individuals. Moreover, the founder boar at the age of 2 years and 10 months had an even lower enzyme amount in liver, kidney and spleen and higher HS storage in all tested tissues compared with the *NAGLU^+/−^* F_1_ pigs at the age of 180 days (Fig. 2C,D). We also conducted immunohistochemical staining on brain sections from two randomly selected 2.5-year-old *NAGLU^+/−^* pigs and two WT half-sibs. *NAGLU* expression tended to decrease in the brain tissue from the *NAGLU^+/−^* pig compared with the normal half sib (Fig. 3). It should be noted that the near absence of NAGLU in the brain of *NAGLU^+/−^* pigs does not correlate with an absence of the protein *in vivo*. It is more likely the case that the reduced protein expression went below the detection limit in our experiment.

### Gross phenotypes of *NAGLU^+/−^* pigs

We obtained 22 F_1_ pigs only carrying the insertion site at the *NAGLU* locus and 22 F_1_ individuals that harbor both insertion sites at the *NAGLU* and *CMTM8* loci. These 44 animals were all *NAGLU^+/−^* pigs, because the insertion of the exogenous gene has no effect on the expression of *CMTM8*. Of the 44 pigs, eight were intentionally slaughtered for sample collection, 20 died within a year of birth including seven that died of severe diarrhea before the age of 21 days. Twelve lived between 1 and 2 years, four between 2 and 3 years and none survived for over 3 years. The founder boar lived 2 years and 10 months, when he became emaciated, motionless and had difficulty standing (Fig. S1). All littermate or half-sib *NAGLU^+/+^* pigs lived normally. Eight (18.2%) of the 44 *NAGLU^+/−^* pigs developed dewclaws (suspended hoof) (Fig. S2).

**Fig. 4.**
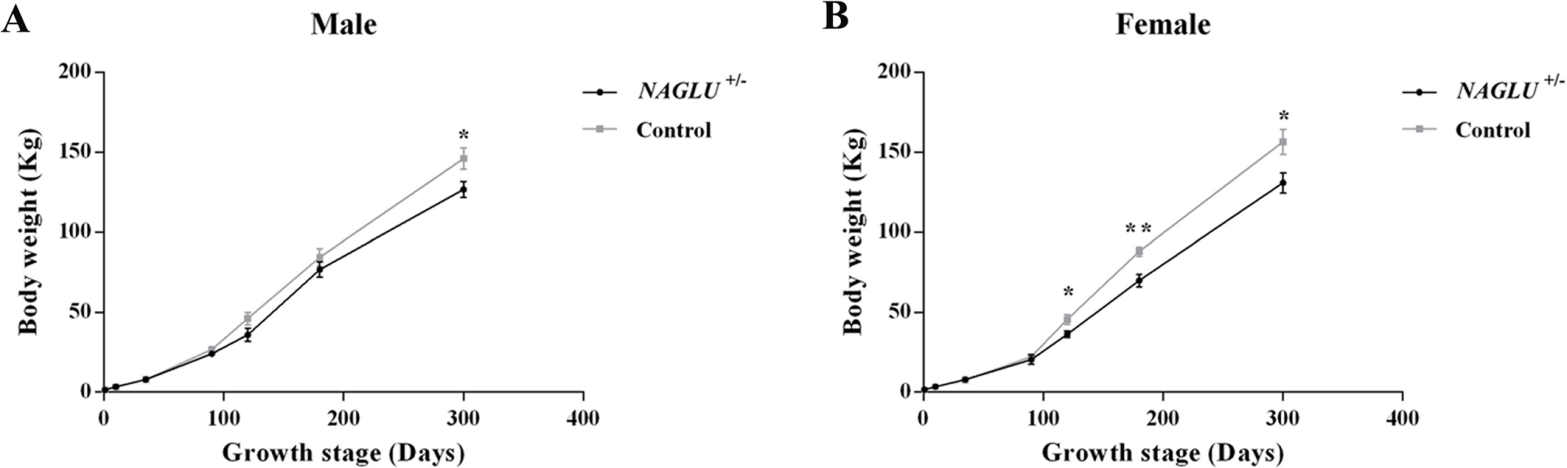
**Visual and growth phenotypes of *NAGLU*^+/−^ pigs and their wild-type half sibs.** (A) Growth curves of *NAGLU***^+/−^** (*n*=35) and half-sib control (*NAGLU***^+/+^**) (*n*=35) boars. (B) Growth curves of *NAGLU***^+/−^** (*n*=35) and half-sib control (*NAGLU***^+/+^**) (*n*=35) sows. Data are presented as mean±s.e. *P<0.05; **P<0.01.

A significant growth retardation of *NAGLU^+/−^* boars was observed at the age of 300 days, when the average body weight of *NAGLU^+/−^* boars (126.74±4.99 kg) was approximately 87% of that of WT (*NAGLU^+/+^*) half sibs (146.11±6.77 kg; *P*<0.05; Fig. 4A). The growth lag was more notable in sows. *NAGLU^+/−^* sows had 80% and 83% of body weight of their WT half sibs from age of 120 days to 300 days (*P*<0.05; Fig. 4B).

### Hematological parameters of *NAGLU^+/−^* pigs

Blood routine examination was performed on *NAGLU^+/**−**^* pigs in comparison with half-sib controls at ages of 150, 200 and 250 days (Tables S1-S3). A higher ratio of monocyte (% of WBC) was found in 150-day-old *NAGLU^+/−^* pigs (16.89±4.49 in *NAGLU*^+/−^ pigs versus 4.58±1.59 in controls, *P*<0.05, Table S1). A significant elevation of lymphocyte count and ratio of lymphocyte were observed in 200-day-old *NAGLU^+/−^* pigs (lymphocyte count: 15.92±1.29 in *NAGLU^+/−^* pigs versus 11.05±0.77 in controls, *P*<0.01; ratio of lymphocyte: 77.42±3.96 in *NAGLU^+/−^* pigs versus 60.89±4.12 in controls, *P*<0.01; Table S2). Moreover, a higher monocyte count was found in 250-day-old *NAGLU^+/−^* pigs (4.92±0.92 in *NAGLU^+/−^* pigs versus 2.14±0.78 in control, *P*<0.05, Table S3). No significant difference (*P*>0.05) in the other hematological parameters was found between *NAGLU^+/−^* and *NAGLU^+/+^* pigs at any of the three ages (Tables S1-S3).

### Visual inspection of internal organs and histopathology

Two *NAGLU^+/−^* pigs were randomly selected and slaughtered for inspection at each of the ages of 1, 180 and 240 days. No abnormality was found in 1-day-old newborn pigs, while all the older pigs had hepatomegaly (Fig. S3A). One 180-day-old pig and one 240-day-old pig had severe pleural adhesions (Fig. S3B) along with apparent lung shrinkage (Fig. S3C) and the younger one also had abnormal pericardium (Fig. S3D). Necropsy of the founder boar revealed pathological changes in the lung, heart, liver, spleen and kidney (Fig. S1).

**Fig. 5.**
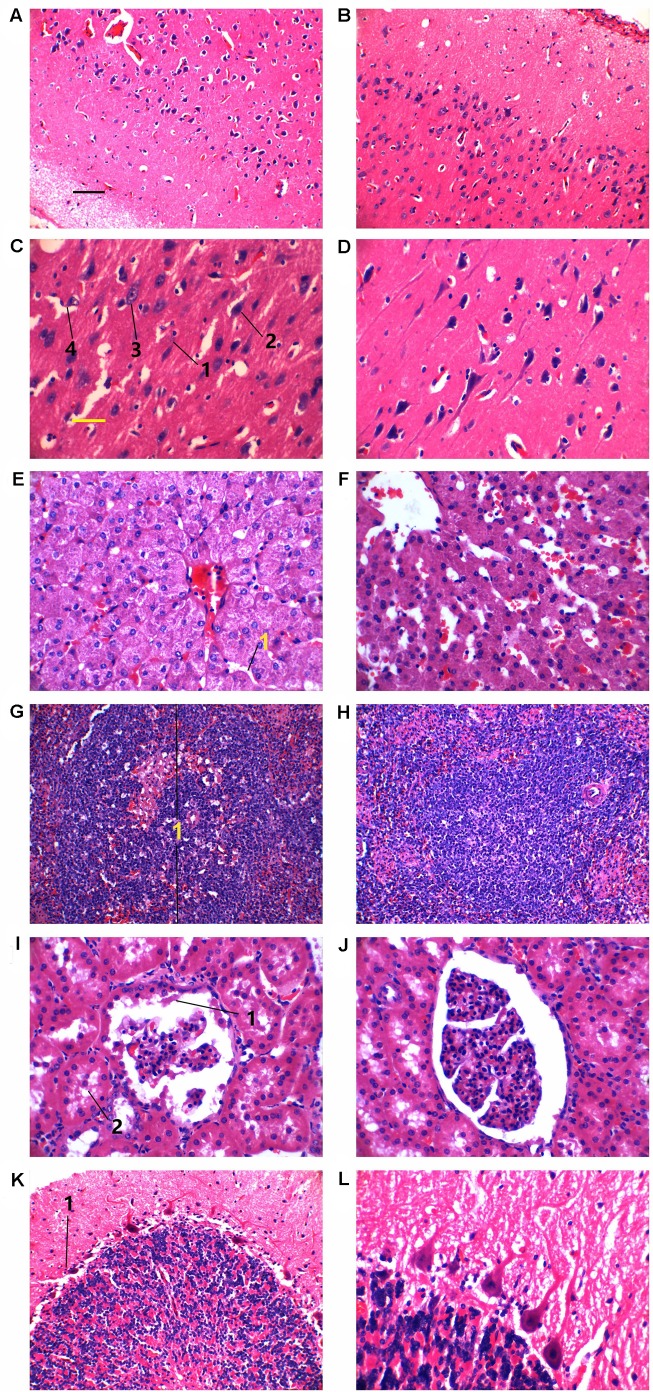
**Hematoxylin and Eosin staining of tissues from two *NAGLU*^+/−^ pigs and their two wild-type half sibs at the age of 240 days.** (A-D) Cerebra. (E,F) Liver. (G,H) Spleen. (I,J) Kidney. (K,L) Cerebella. In C: 1, shortened pyramidal cells; 2, nuclei in neurons appeared shrunk and stained darker; 3, vacuole formation; 4, mild microgliosis. In E: 1, narrow hepatic sinusoid. In G: 1, one splenic corpuscle. In I: 1, hyaline droplets; 2, albumin leakage. In K: 1, one purkinje neuron. Left panels, *NAGLU***^+/−^** pigs. Right panels, wild-type half sibs. Black scale bar for A,B,G,H: 50 µm. Yellow scale bar for C-F,I-L: 25 µm.

In addition to these six *NAGLU^+/−^* pigs, two of their *NAGLU^+/+^* half sibs were also slaughtered at each of the three ages for histology comparison. Haemotoxylin and Eosin (H&E) staining was performed on cerebrum, cerebellum, liver, spleen and kidney sections (Fig. 5; Fig. S4). All tissues of 1-day-old pigs appeared normal (data not shown). In the cerebrum sections of *NAGLU^+/−^* pigs at both ages of 180 and 240 days, the molecular layer, granular layers and pyramidal layers appeared generally well organized, but dissolution of white matter was evident (Fig. 5A; Fig. S4A) compared with the control groups (Fig. 5B; Fig. S4B). Dendrites of pyramidal cells were apparently shorter (4±0.06 μm, Fig. 5C; 5±0.12 μm, Fig. S4C) than those in controls (19±0.30 μm, Fig. 5D; 20±0.20 μm, Fig. S4D), and nuclei in neurons stained darker. Nuclei were missing in some neurons, along with vacuole formation (Fig. 5C-3; Fig. S4C-3), and mild microgliosis and neuronophagia existed (Fig. 5C-4; Fig. S4C-4).

H&E staining showed distorted hepatocyte plates and cramped sinusoids in the liver due to swelling of some hepatocytes in *NAGLU^+/−^* pigs; vacuoles and granular matter that stained denser were frequently observed in cells. Kupffer cells were more frequently found in *NAGLU^+/−^* pigs (Fig. 5E-1; Fig. S4E-1) than in controls (Fig. 5F; Fig. S4F). Architecturally, there was no deformation in the spleen sections of *NAGLU^+/−^* pigs (Fig. 5G; Fig. S4G) compared with *NAGLU^+/+^* pigs (Fig. 5H; Fig. S4H). Splenic corpuscles appeared larger and a few enclosed lymphocytes had condensed nuclei in *NAGLU^+/−^* pigs (Fig. 5G-1; Fig. S4G-1). Kidney sections from *NAGLU^+/−^* pigs showed stainless, tear-shaped structures inside the Bowman's capsules (Fig. 5I-1; Fig. S4I-1), which were most likely albumin. Albumin leakage was observed in the proximal convoluted tubules (Fig. 5I-2; Fig. S4I-2), where scattered vacuoles were also present as compared to WT pigs (Fig. 5J; Fig. S4J). Staining of cerebellum did not show fundamental pathological changes in *NAGLU^+/−^* pigs (Fig. 5K) at 240 days of age compared with *NAGLU^+/+^* pigs (Fig. 5L), and, notably, Purkinje neurons were intact (Fig. 5K-1).

### MRI results

**Fig. 6.**
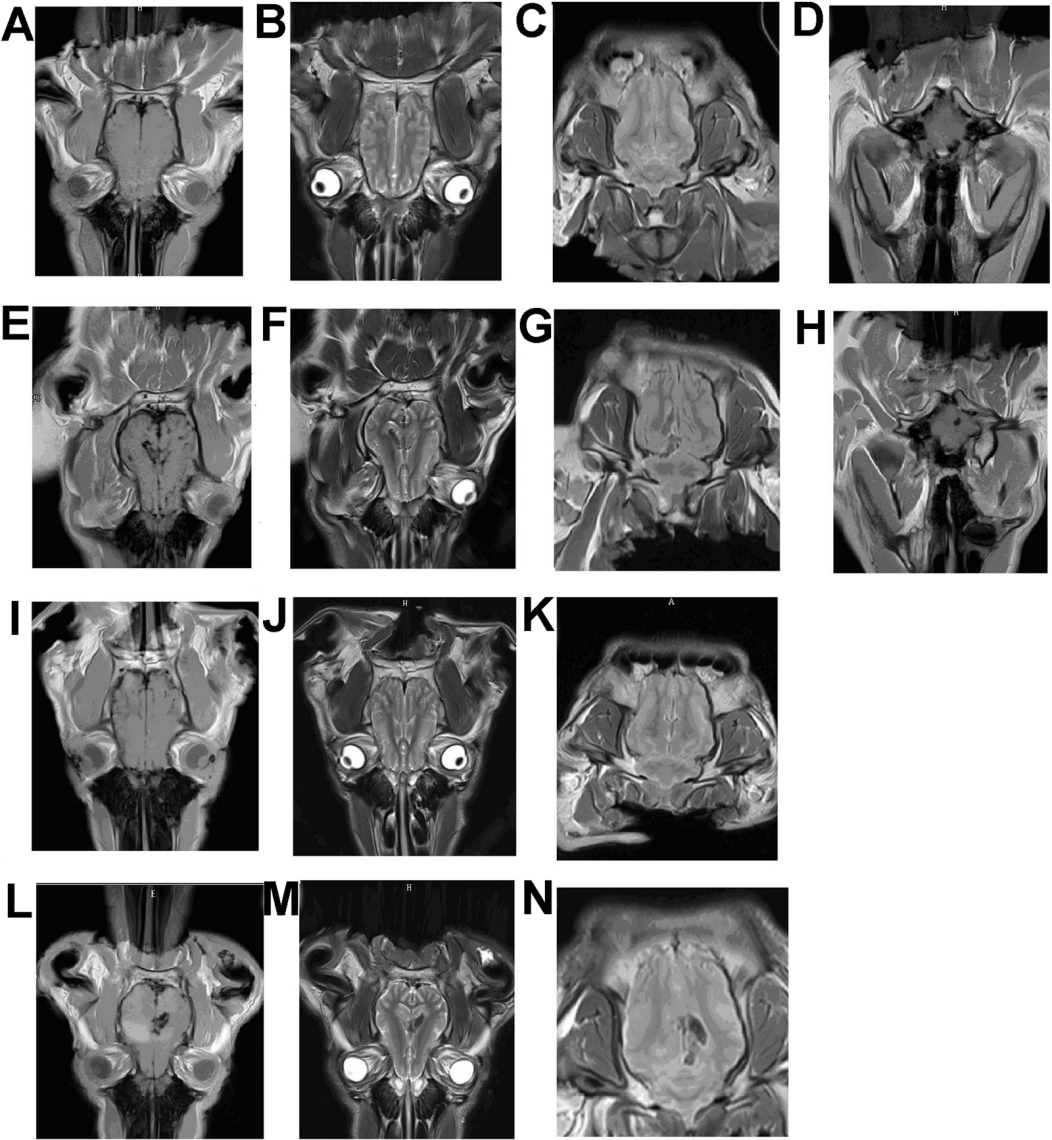
**Magnetic resonance imaging of pigs at the age of 180 days.** (A-D) One wild-type pig. (E-N) Three NAGLU^+/−^ pigs. (A,E,I,L) Axial T1-weighted images of brain. (B,F,J,M) Axial T2-weighted images of brain. (C,G,K,N) Fluid-attenuated inversion recovery images of brain. (D,H) Axial T1-weighted images of cerebellar.

To provide guidelines for future studies, we examined MRI characteristics of one randomly selected normal pig (Fig. 6A-D) and three *NAGLU^+/−^* pigs at the age of 180 days (Fig. 6E-N). Gross cortical atrophy of varied severities was evident in all three *NAGLU^+/−^* pigs. Diverse pathological features were noted in the left intracerebral capsule, the parietal lobes, the thalamus and cerebellar of these *NAGLU^+/−^* pigs. High T1 (Fig. 6E) and low T2 (Fig. 6F) signals were observed in the brain of one *NAGLU^+/−^* pig. The fluid-attenuated inversion recovery image presented a slightly low signal in this area (Fig. 6G). Oval-shaped high signal spots were in the vicinity of cerebellopontine angle of the left hemisphere in T1-weighted image (Fig. 6H). One *NAGLU^+/−^* pig showed widening sulcus and fissures of both hemispheres of the brain, and exhibited a few high signal areas in the right temporal lobe and in the thalamus in the axial T1-weighted image (Fig. 6I). No significant abnormality was found in T2-weighted and fluid-attenuated inversion recovery images of brain for this pig (Fig. 6J-K). In another *NAGLU^+/−^* pig, widening sulcus and fissures of both hemispheres of the brain were also found, and a high T1 signal patch affecting the temporal lobes of both sides was observed (Fig. 6L). In addition, this pig showed enlargement, high T1 and low fluid-attenuated inversion recovery signals in the left internal capsule (Fig. 6L-N). No obvious pathological feature was found in T2-weighted image of brain (Fig. 6M) for this pig, and no evident pathological changes in T1-weighted images of cerebellar was found in two *NAGLU^+/−^* pigs (data not shown).

### CT scans and X-ray imaging

CT scans of the three 180-day-old *NAGLU^+/−^* pigs indicated cerebral atrophy of varied degrees in the brains. Fig. S5 shows a CT image in comparison with that of a littermate *NAGLU^+/+^* pig. X-ray imaging did not show obvious bone structural aberration in any of the four limbs (Fig. S6).

## DISCUSSION

### NAGLU enzyme amount and HS storage of *NAGLU^+/−^* pigs resemble those of MPS IIIB affected patients and animals

The most typical characteristics of MPS IIIB affected patients (Yogalingam et al., 2000) and animal models (Ellinwood et al., 2003; Li et al., 1999) include decrease of NAGLU enzyme and accumulation of HS. In this study, we tested the amount of NAGLU enzyme and HS accumulation in *NAGLU^+/−^* F_1_ pigs and the founder boar. We show that the NAGLU enzyme amount is significantly decreased (Fig. 2C, *P*<0.05) while the HS storage is remarkably increased (Fig. 2D, *P*<0.05) in *NAGLU^+/−^* F_1_ pigs compared with WT pigs. The older founder boar had an even lower enzyme amount and higher HS storage (Fig. 2C,D) than younger *NAGLU^+/−^* F_1_ pigs (2.8 versus 0.5 years of age, respectively). Moreover, we conducted immunohistochemical staining in the brain sections from two randomly selected *NAGLU^+/−^* and two WT pigs at the age of 2.5 years. We observed reduced NAGLU protein in the brain tissue of *NAGLU^+/−^* pigs (Fig. 3).

### Growth phenotypes and pathology of *NAGLU^+/−^* pigs reflect characteristics of MPS IIIB affected patients and animals

Coarse and altered facial features are common in MPS IIIB affected patients and animals, and growth retardation is another frequently observed phenomenon at later stages (Li et al., 1999; Karageorgos et al., 2007; Barone et al., 2001). *NAGLU*-knockout mice had shorter snouts (Gografe et al., 2003), and one MPS IIIB affected dog experienced coat color change from black to deep auburn (Ellinwood et al., 2003). The skin and hair coat of *NAGLU^+/−^* pigs (Large White) remain unchanged (solid white) in our study, and no obvious facial deformation was observed in all *NAGLU^+/−^* pigs except that dewclaws were found in eight individuals. Humans – as MPS IIIB patients often show pathological changes in the skeleton at late stage – may suffer from difficulties in standing and walking due to muscle cramps, stiff joints, or even aseptic necrosis of the femoral head (Barone et al., 2001). In this study, X-ray imaging did not show apparent skeletal deformation in *NAGLU^+/−^* pigs at the age of 180 days (Fig. S6). But considering that our observation was mostly on young pigs, it would be more informative to examine the skeletal changes in the pigs at advanced stages of the disease in the near future. Indeed, the 2.8-year-old founder had difficulty standing and eight *NAGLU^+/−^* pigs at the age of 1-1.5 year developed dewclaws, likely due to skeletal deformation. In this study, growth of the *NAGLU^+/−^* pigs was retarded compared to controls, as often seen in humans and other animals with MPS IIIB.

Few studies have reported blood parameters of MPS IIIB patients or animals. MPS IIIB affected dogs had elevated counts of blood platelets, lymphocytes and basophils (Ellinwood et al., 2003). In this study, *NAGLU^+/−^* pigs at the ages of 150, 200 and 250 days did not have a consistent trend in blood parameters. For example, compared with the *NAGLU^+/+^* pigs, 150-day-old and 250-day-old, but not 200-day-old, *NAGLU^+/−^* pigs had a higher monocyte ratio. For lymphocyte count and lymphocyte ratio, higher values were observed in 200-day-old *NAGLU^+/−^* pigs, but not in 150-day-old and 250-day-old *NAGLU^+/−^* pigs (Tables S1-S3). For the other hematological parameters, no significant difference was found between *NAGLU^+/−^* and *NAGLU^+/+^* pigs. This indicates that routine blood examination may be not a suitable diagnostic tool for porcine MPS IIIB.

Mortality often occurs at around 25 years of age in human patients preceded by worsening spasticity, joint stiffness, immobility, unresponsiveness and swallowing difficulties. Patients may die of pneumonia, pancreatitis, postoperative complications, heart failure or sudden death (Weber et al., 1999). Sudden death was also found in MPS IIIB affected juvenile emus (Palmieri et al., 2015). In this study, the average life span of *NAGLU^+/−^* pigs was 1.5 years, 43% of *NAGLU^+/−^* pigs died younger than 1 year and only one pig lived close to the age of 3 years, indicating that these *NAGLU^+/−^* pigs resemble a severe type of human MPS IIIB. Most *NAGLU^+/−^* pigs died suddenly, and symptoms at death in all pigs included depression, flatulence, fever and cramping. Functional deterioration of tissues and organs by the accumulation of heparin sulfate may have led to high susceptibility in these pigs to various pathogens, hence varied symptoms at death. Autopsy of four *NAGLU^+/−^* pigs that experienced sudden death revealed pronounced pathological changes in the lungs (Fig. S7), indicating high susceptibility of this organ, which is in agreement with one previous report that the most common cause of death in MPS IIIB patients was pneumonia (Valstar et al., 2010).

### Histopathology of *NAGLU^+/−^* pigs mimics that of MPS IIIB affected patients and animals

Though brain damage is the most common feature in all MPS IIIB patients and affected animal models, disorder in the liver, kidney and lungs have also been reported in many cases (Li et al., 1999; Karageorgos et al., 2007; Yogalingam et al., 2000; Palmieri et al., 2015; Garbuzova-Davis et al., 2009; Moog et al., 2007). Accumulation of glycosaminoglycans is the main cause of vacuole formation and degeneration of neurons, and loss of Purkinje cells is common in patients. Deterioration of the central nervous system leads to progressive dementia, gait abnormality and impaired vision and hearing (Ellinwood et al., 2003; Valstar et al., 2010). Hyperphosphorylated tau was found in certain neurons in a mouse model of MPS IIIB but not reported in human patients or other animal models (Ohmi et al., 2009), thus whether a connection between MPS IIIB and tauopathy exists is not clear. Purkinje cell loss, ballooning of Purkinje cells and swelling of molecular layer dendrites are typical pathology findings in MPS IIIB (Ellinwood et al., 2003; Li et al., 1999; Karageorgos et al., 2007). The *NAGLU^+/−^* pigs, at both 180 and 240 days of age, manifested vacuolization in neurons and microglia along with mild neuronophagia, but loss or alteration of Purkinje cells was not evident (Fig. S4). These features are very similar to a case of 17-year-old patient whose cerebellum was also nearly intact (Hadfield et al., 1980).

Hepatomegaly (Ellinwood et al., 2003; Valstar et al., 2010; Champion et al., 2010) and hepatocyte vacuolization (Li et al., 1999; Karageorgos et al., 2007) are common in MPS IIIB, which were also observed in the histological study of *NAGLU^+/−^* pigs. Granule formation was observed in liver cells of *NAGLU^+/−^* pigs (Fig. 5E). In the kidney, vacuolization was not apparent in the lining endothelial cells of the glomerulus but was seen in the proximal convoluted tubules, and transparent tear-shaped objects, likely albumin droplets, were observed in the proximal convoluted tubules and in the Bowman's capsules. Albumin leakage, if true, was probably a result of defective filtration due to GAG accumulation. There was no obvious splenomegaly in *NAGLU^+/−^* pigs, and histology did not reveal significant abnormality except for slightly larger splenic corpuscles.

### MRI characteristics of *NAGLU^+/−^* pigs

MRI examinations often show symptoms of hydrocephalus and spinal cord compression that would be indicated by low T1- and T2-weighted signals in MPS IIIB affected patients; ventricular dilatation and cerebral atrophy were often indicated (Neufeld and Muenzer, 1995; Kendall, 1992). T2-weighted images often indicate demyelination, glial cell hyperplasia and hydrocephalus (Zafeiriou et al., 2001). MRI images of *NAGLU^+/−^* pigs showed ventricular dilatation and cerebral atrophy, but not hydrocephalus. MRI findings may vary among patients, and features for the same patient may also change with age and disease progression (Zafeiriou et al., 2001). Our study also showed variations among the three *NAGLU^+/−^* pigs from the same litter, thus cautions need to be taken when using MRI for diagnosis or evaluation of MPS IIIB disease stages.

### Possible mechanism of MPS IIIB in *NAGLU^+/−^* pigs

**Fig. 7.**
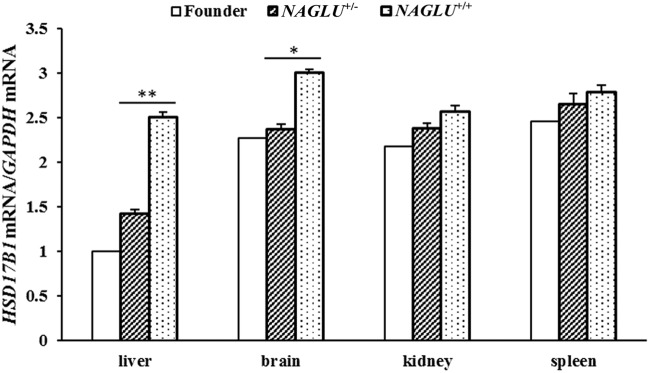
**The mRNA levels of *HSD17B1* in four tissues from the 2.8-year-old founder and six 180-day-old F_1_ pigs.** *NAGLU***^+/−^**, F_1_ pigs that carry one insertion site at the *NAGLU* locus (*n*=3). *NAGLU***^+/+^**, wild-type pigs (*n*=3). Data are presented as mean±s.e. except the founder boar. *P<0.05; **P<0.01. Phenotypic difference was not evaluated between the founder and the other individuals as only one founder animal was tested.

A high degree of genetic heterogeneity was found in MPS IIIB patients, in which more than 100 mutations were associated with this disease (Valstar et al., 2008). Mice with a homozygous disruption of exon 6 of the *NAGLU* gene are viable with MPS IIIB phenotypes at advanced age (Li et al., 1999). As mentioned above, *NAGLU^+/−^* pigs showed phenotypes of MPS IIIB, which is likely due to the reduced *NAGLU* expression. It is interesting that the disruption of one allele causes MPS IIIB in pigs. To understand the differences between the outcome of pigs and mice, we made a close examination on the disruption site in *NAGLU^+/−^* pigs and *NAGLU^−/−^* mouse. In the *NAGLU^−/−^* mouse, the distance between the disruption site of the *NAGLU* gene and the neighboring *HSD17B1* gene is larger than 1 kb (Li et al., 1999; Di Natale et al., 2005), while it is only 321 bp between the insertion site of the exogenous gene (*BMPR-IB*) and the transcription start site of *HSD17B1* in *NAGLU^+/−^* pigs. It is thus possible that the insertion in pigs destroys the transcription regulator of the *HSD17B1* gene and causes the dysfunction of this gene. To test this possibility, we measured the *HSD17B1* mRNA level in tissues from 180-day-old *NAGLU^+/−^* pigs via qRT-PCR. The expression levels of *HSD17B1* were significantly decreased in liver (*P*<0.01) and brain (*P*<0.05) from the *NAGLU^+/−^* pigs compared with those from WT (*NAGLU^+/+^*) pigs (Fig. 7). Thus, we assume that the deficiencies of *NAGLU* and *HSD17B1* could exert a dosage effect and synergistically induce severe MPS IIIB phenotypes of *NAGLU^+/−^* pigs in our study. In mice, a study reported that deleting the *HSD17B1* gene results in a hypomorphic Naglu allele and a phenotype mimicking a lysosomal storage disease (Jokela et al., 2017). It should be mentioned that pigs homozygous for the disruption of the *NAGLU* gene (*NAGLU^−/−^*) are embryonic lethal (data not shown), which may be due to the collective effects of the deficiencies of *NAGLU* and *HSD17B1*. Further studies are required to test this hypothesis.

## CONCLUSIONS

In summary, we demonstrates that pigs carrying a heterozygous disruption of the *NAGLU* gene (*NAGLU^+/−^*) develop typical symptoms of MPS IIIB and thus represent a model of MPS IIIB. To our knowledge, this is the first report of a porcine model for MPS IIIB. Pig models are desirable in many pre-clinical studies as they are large, have similar physiological and pathological features to human patients, and the use of a pig model bears fewer ethnic burdens than using primates and dogs. Thus, these *NAGLU*^+/−^ pigs represent an ideal model for the study of MPS IIIB.

## MATERIALS AND METHODS

### Ethics statement

All experiments that involved animals were carried out in accordance with the approved guidelines by the Ministry of Agriculture of China. Approval was obtained from the ethics committee of Jiangxi Agricultural University before the experiment.

### Animals

The founder boar mated with seven non-transgenic Large White (Canada) sows, generating 92 F_1_ live piglets. All pigs were raised on a farm in Jinxi County, Jiangxi Province, China. Piglets were weaned at the age of 28 days and then fed with a feed containing 18.8% crude protein, 3.4% crude fiber, 5.1% crude ash, 0.9% calcium and 0.72% total phosphorus. At 60 days of age, pigs were supplied with another feed containing 16.9% crude protein, 5.3% crude fiber, 6.4% crude ash, 0.85% calcium and 0.56% total phosphorus. Pigs were vaccinated for circovirus, streptococcus, classical swine fever virus, blue-ear virus, picornavirus (foot and mouth disease), pseudorabies and classical swine fever virus at the ages of 10, 15, 21, 28, 35, 42 and 50 days, respectively.

### PCR genotyping

Genomic DNA was extracted from ear samples using phenol/chloroform method. Primers F1/R1 (Table S4) were implemented to identify transgenic and non-transgenic (WT) pigs as previously reported (Zhao et al., 2016). Primers F2/R2 and F3/R3 (Table S4) were used to classify the transgenic individuals with one or two insertion sites. PCR was performed in a routine way at optimal annealing temperatures (Table S4). PCR products were separated in 0.8-1.5% agarose gels and sequenced using a 3130XL Genetic Analyzer (Applied Biosystems, USA).

### Quantitative real-time PCR (qRT-PCR)

Total RNA was isolated from liver, brain, kidney and spleen tissues that were collected from three *NAGLU*^+/−^ individuals and three WT pigs at the age of 180 days using TRIzol (ABI 15596018, USA). cDNA was synthesized using the PrimeScript RT reagent Kit with gDNA Eraser (TaKaRa RRO47A, Japan). The expression levels of *NAGLU*, *CMTM8* and *HSD17B1* were tested using SYBR Premix EX Taq II (Tli RNaseH Plus) (TaKaRa RR820A, Japan) on a 7900HT Fast Real-Time PCR System (ABI, USA). Porcine *GAPDH* was used as an internal control. Each reaction was performed in triplicate (technical replicates). The relative quantification of *NAGLU* was calculated by the 2^−ΔΔCt^ method. Primer pairs for qRT-PCR of *NAGLU*, *CMTM8*, *HSD17B1* and *GADPH* were F4/R4, F5/R5, F6/R6 and F7/R7, respectively (Table S4).

### ELISA assay

Liver, brain, kidney and spleen tissues for qRT-PCR analysis were also used for ELISA assay. These tissues were homogenized in the PBS (phosphate buffered saline) buffer. The homogenized samples were frozen-thawed twice in −80°C refrigerator and then the protein concentration of supernatants was determined using the BCA Protein Assay Kit (Beyotime Biotechnology P0010, China). The samples were stored for detecting enzyme amount and GAG amount.

#### Enzyme amount analysis

Enzyme amount was detected according to the protocol of porcine alpha-N-acetyl-glucosaminidase Elisa Kit (Cusabio CSB-EL015416PI, China). Briefly, the standard solutions were diluted from 2 ng/ml to 0.0313 ng/ml based on a specific gradient (2^0^, 2^1^, 2^2^, 2^3^, 2^4^, 2^5^, 2^6^ and 2^7^) using dilution buffer. Standard dilution buffer was used as blank sample. Protein samples were diluted to a final concentration of 100 µg/ml, and 100 µl of each test sample, standard solutions and blank sample were added into the 96-well plate. The plate was incubated at 37°C for 2 h. Then 100 µl of biotinylated detection antibody was added into each well, and the plate was incubated at 37°C for 1 h. Subsequently, 100 µl of HRP-labeled streptavidin working solution was put into each well that was then incubated at 37°C for 1 h. Ninety microliters of 3,3′,5,5′-Tetramethylbenzidine (TMB) substrate was added to each well, and the plate was incubated at 37°C for 15-30 min. Finally, 50 µl of stop solution was put into each well, and a M200 PRO automatic ELISA reader (TECAN, USA) was explored to read the absorbance (OD) at 450 nm. Each reaction was performed in duplicate. The NAGLU enzyme amount was calculated based on the standard curve.

#### Analysis of GAG storage

The GAG HS storage was detected using the Porcine HS ELISA Kit (RenJieBio RJ18537, China) according to the user manual. First, standard solutions were prepared in 50 µl dilution solution to final concentrations of 8, 4, 2, 1, 0.5 and 0.25 µg/ml, respectively. Ten micrograms of each sample was also diluted in 50 µl dilution solution. Dilution solution was set as a blank sample. Then 50 µl of each sample and standard solutions were added to the 96-well plate. Next, 50 µl of enzyme conjugate was put into every well with sample and standard solution, and the plate was incubated at 37°C for 60 min. Subsequently, the plate was washed four times by wash solution. Fifty microliters of substrate A and B were added to each well successively, and the plate was incubated at 37°C for 15 min. Finally, 50 µl of stop solution was added to each well, and the OD value at 450 nm was read using the automatic ELISA reader (TECAN, USA). The HS storage amount was calculated based on the standard curve.

### Immunohistochemical staining

Immunohistochemical staining was performed on brain tissues from two randomly selected 2.5-year-old *NAGLU***^+/−^** pigs and two WT half sibs. Tissues were fixed with 4% paraformaldehyde in 0.1 M phosphate buffer (pH 7.4) for 48 h, paraffin-embedded, and sectioned. Antigen retrieval was performed on deparaffinized sections in 0.01 M citrate buffer (pH 6.0) by the microwave method (Yano et al., 2003). Slides were incubated in 3% hydrogen peroxide in methanol for 30 min to eliminate endogenous peroxidase activity, and blocked with 10% goat serum in phosphate buffered saline (PBS; Gibco, USA) for 1 h. Sections were incubated with 50×diluted rabbit anti-NAGLU antibody (ab72178, Abcam, UK) overnight at 4°C and at room temperature for 40 min on the next day. Slides were washed with PBS three times and incubated with 1000× diluted biotin-conjugated goat anti-rabbit IgG (Kangwei Shiji, China) for 2 h at 37°C. Sections were washed three times with PBS and incubated with HRP-conjugated streptavidin for 1 h at 37°C, washed twice with PBS and once with tris-HCl buffered saline, then incubated with Diaminobenzidine peroxidase substrate solution for 30 min at room temperature, protected from direct light. Slides were then washed and mounted with Canada balsam for microscopic examination. The slides were observed on a BA410 microscope (Motic, China).

### Measurement of blood parameters and growth rate

Blood samples were collected from the marginal ear veins of F_1_ transgenic and WT pigs at ages of 150, 200 and 250 days and were analyzed using a BC-6800 automated blood analyzer (MindRay, China). Body weights of *NAGLU*^+/−^ pigs and their WT half sibs were recorded at birth and at the ages of 1, 10, 35, 90, 120, 180 and 300 days, respectively. At each time point, at least five pigs (all males or females) of each group (*NAGLU*^+/−^ and WT) were recorded. Growth curves were drawn using the recorded data.

### Visual inspection and histological examination of internal organs and tissues

Two *NAGLU*^+/−^ pigs were randomly selected and slaughtered at each of the ages of 1, 180 and 240 days for visual inspection and histological examination. Two littermate WT pigs were used as controls at each age. Pieces of liver, spleen, kidney, cerebrum and cerebellum were fixed using 4% paraformaldehyde in 0.1 M phosphate buffer (pH 7.4) for 48 h. Fixed tissues were embedded in paraffin, and sections were processed and stained with H&E. The H&E slides were observed on a BA410 microscope (Motic, China).

### CT scan, MRI and X-ray imaging

Three *NAGLU*^+/−^ pigs and one WT half sib were randomly selected and slaughtered at the age of 180 days. Their heads were immediately sent to the First Affiliated Hospital of Nanchang University in refrigerated containers for MRI on a 1.5T MRI machine (Philips, The Netherlands) and CT scan on a Somatom Emotion 16 scanner (Siemens, Germany). Parameters for MRI were: THK=1 mm, TR=1900 ms, TE=2.27 ms, TI=900, ETL=1k/sapce, FA=9.0°. Parameters for CT were: FOV=241 mm, THK=1 mm. In addition, X-ray images were obtained for fore and hind legs of the three *NAGLU*^+/−^ pigs and one WT half sib to examine the bone architecture in the limbs.

### Statistical analyses

The statistical analysis was performed by Student's *t*-test in SPSS 17.0 (Statistical Package for the Social Sciences). *P*<0.05 was considered significant and *P*<0.01 was considered highly significant.

## Supplementary Material

Supplementary information

First Person interview
