# Supplementary material for: A model of mucopolysaccharidosis type IIIB in pigs
Source: Biol Open. 2018 Sep 26;7(10):bio035386. doi: 10.1242/bio.035386 (PMC6215415; doi:10.1242/bio.035386)
Supplement: Supplementary information [file biolopen-7-035386-s1.pdf]

FIRST PERSON

First person – Qiang Yang

First Person is a series of interviews with the first authors of a selection of papers published in Biology Open, helping early-career researchers promote themselves alongside their papers. Qiang Yang is co-first author on 'A model of mucopolysaccharidosis type IIIB in pigs', published in BiO. Qiang is a PhD student in the lab of Dr Lusheng Huang at Jiangxi Agricultural University, Nanchang, China, investigating the improvement of economically important traits in pigs through genome editing using the CRISPR/Cas9 technique.

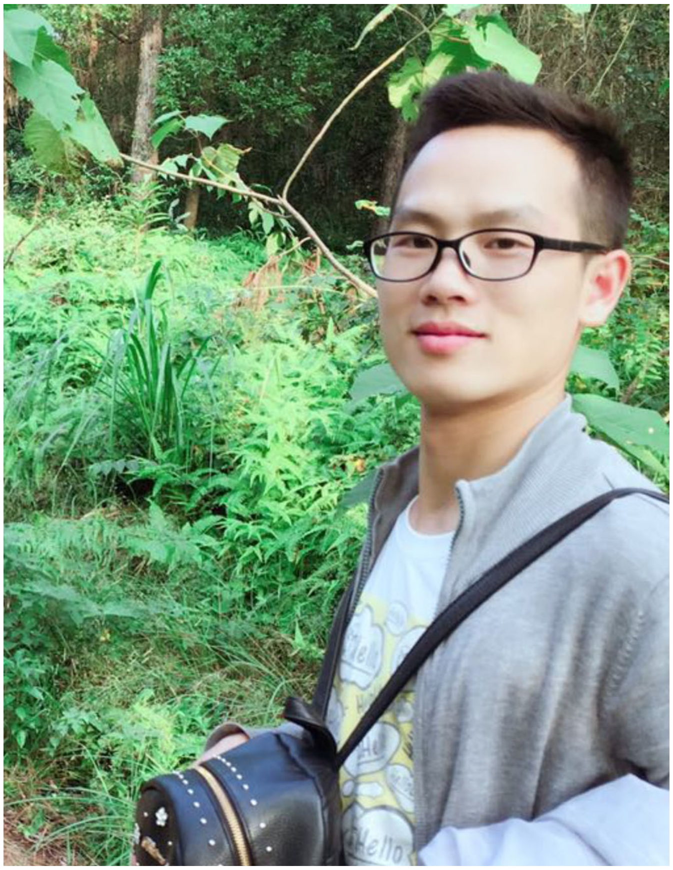

Qiang Yang

What is your scientific background and the general focus of your lab?

I hold a bachelor's degree in animal sciences and I am currently a doctoral graduate student in a program in animal genetics, breeding and reproduction. My research focuses on the improvement of economically important traits in pigs through genome editing using the CRISPR/Cas9 technique. Our laboratory has been making great efforts towards uncovering the genetic groundwork for economically important traits and diseases in farm animals, especially pigs.

How would you explain the main findings of your paper to non-scientific family and friends?

Mucopolysaccharidosis type IIIB (MPS IIIB) is a rare metabolic disorder caused by mutations in the *NAGLU* gene. Individuals with this disease do not have sufficient NAGLU enzyme to degrade heparan sulfate, a type of polysaccharide that performs various functions in animal tissues. This causes accumulation of heparan sulfate in lysosomes, a part of the cell that acts as a waste-disposal system, and eventually leads to the malfunction of these critical organelles. An important reason that the disease is rare is that it is vicious: only patients who have non-critical *NAGLU* mutations (thus slower tissue and organ degeneration) can live to adulthood. In our study, pigs missing one of the two copies of the *NAGLU* gene developed typical symptoms of human MPS IIIB. Pigs are an ideal large animal model for human diseases due to their similarities to humans in physiological and pathological features, and we report here the first pig model for research into MPS IIIB.

What are the potential implications of these results for your field of research?

Pig models are desirable in many preclinical investigations as they have similar proportional organ sizes and similar physiological and pathological features to those of human patients. Our pig model for MPS IIIB displays a pathogenic condition of lowered levels of the NAGLU enzyme, and our detailed characterization of this model lays the groundwork for better understanding of the disease and the search for potential therapeutics.

What has surprised you the most while conducting your research?

This NAGLU knockout allele was obtained when we tried to knock in a desired gene to the porcine genome using the old-fashioned random insertion method. This particular founder pig apparently had an insertion of a broken piece of the transgene at an interesting site: the insertion knocked out the *NAGLU* gene. This was not planned but, to our surprise, it turned out to be a good model for MPS IIIB. This is the amazing nature of scientific research: you often get a whole new opportunity from what seemed to be a failure.

> "This is the amazing nature of scientific research: you often get a whole new opportunity from what seemed to be a failure."

What, in your opinion, are some of the greatest achievements in your field and how has this influenced your research?

Genetic modifications have been done for several decades in a way that is best described as 'a shot in the dark'. This is not the case any

Qiang Yang's contact details: State Key Laboratory of Pig Genetic Improvement and Production Technology, Jiangxi Agricultural University, Nanchang 330045, China.
E-mail: 626818972@qq.com

Biology Open (2018) 7, doi:10.1242/bio.035386: Supplementary information

more, thanks to the development of the CRISPR/Cas system, which makes it possible to achieve genetic modifications in a precise and predictable manner. In my opinion, this rapidly developing technique will have broad applications in many fields such as yield or quality improvement of crops and farm animals through genetic modification, genetic disease corrections, infectious disease prevention, and so on.

What changes do you think could improve the professional lives of early-career scientists?

First of all, young scientists need a broader scope of training, which is hard to get nowadays as scientific disciplines are so finely divided and specialized. Institutes that provide training should encourage trainees to try new techniques rather than 'let them do what they are good at, so we can move forward fast'. This can be more costly, but it is of long-term benefit to young researchers. Early-stage scientists also face difficulties in obtaining research funding. Giving funding preference, or allocating dedicated funds, to starter scientists would greatly improve their professional development. This involves financial policies, and it is done better in some counties than in others. Senior-mentored junior research programs would be another way of assisting the growth of young scientists, I think, and should be highly advocated.

What's next for you?

My graduate research has been progressing quite well and I will earn my PhD degree soon. I feel that I need to broaden the scope of my knowledge and improve my capability for independent research through postdoctoral training. Genome editing is a magical tool and I think I will continue to use it for my immediate future research.

Reference

Yang, Q., Zhao, X, Xing, Y., Jiang, C., Jiang, K., Xu, P., Liu, W., Ren, J. and Huang, L. (2018). A model of mucopolysaccharidosis type IIIB in pigs. *Biol. Open* **7**, bio035386.

Biology Open
